# mRNA as novel technology for passive immunotherapy

**DOI:** 10.1007/s00018-018-2935-4

**Published:** 2018-10-17

**Authors:** Thomas Schlake, Andreas Thess, Moritz Thran, Ingo Jordan

**Affiliations:** CureVac AG, Paul-Ehrlich-Str. 15, 72076 Tübingen, Germany

**Keywords:** mRNA technology, Antibody, Immune globulin, Adoptive cell transfer, CAR T cell, Passive immunization, mRNA design, Tolerability

## Abstract

While active immunization elicits a lasting immune response by the body, passive immunotherapy transiently equips the body with exogenously generated immunological effectors in the form of either target-specific antibodies or lymphocytes functionalized with target-specific receptors. In either case, administration or expression of recombinant proteins plays a fundamental role. mRNA prepared by in vitro transcription (IVT) is increasingly appreciated as a drug substance for delivery of recombinant proteins. With its biological role as transient carrier of genetic information translated into protein in the cytoplasm, therapeutic application of mRNA combines several advantages. For example, compared to transfected DNA, mRNA harbors inherent safety features. It is not associated with the risk of inducing genomic changes and potential adverse effects are only temporary due to its transient nature. Compared to the administration of recombinant proteins produced in bioreactors, mRNA allows supplying proteins that are difficult to manufacture and offers extended pharmacokinetics for short-lived proteins. Based on great progress in understanding and manipulating mRNA properties, efficacy data in various models have now demonstrated that IVT mRNA constitutes a potent and flexible platform technology. Starting with an introduction into passive immunotherapy, this review summarizes the current status of IVT mRNA technology and its application to such immunological interventions.

## Introduction

Our bodies are continuously exposed to molecules that may indicate disease or parasitic invasion. The immune system is responsible for the detection and clearance of such molecules and for control of the underlying causes. Immediate discrimination between self and foreign upon first exposure is mediated by innate immunity. Signals that induce innate immunity are called pathogen-associated and damage-associated molecular patterns (PAMPs and DAMPs) [1, 2]. The innate immune system is characterized by induction of generic defenses against a broad spectrum of infectious agents and is mainly aimed at clearance of tissue damage at the site of infection and interruption of further pathogen replication. Responses against specific targets following prolonged or repeated exposures are processed by the adaptive immune system [3]. Important effectors of adaptive immunity are highly specialized cells: B cells secrete antibodies against soluble or cell-associated antigens [4]. Cytotoxic CD8^+^ T cells (CTLs) recognize and kill infected or neoplastic cells [5]. Regulatory CD4^+^ T cells augment B cell maturation or inhibit auto-reactive immune cells [6]. Dendritic cells (DCs) process and present antigen for the transition from innate to adaptive immunity [7]. Some B and T cells progress towards persisting memory cells that react faster and with greater affinity to the foreign molecules upon re-exposure. Compared to innate immunity, adaptive immunity usually requires weeks (as opposed to minutes) until an effective response against novel targets is achieved. Advantages of the adaptive immune system include the ability to launch specific immune responses also against antigens of endogenous (not only microbial) origin that may be associated with degenerative or neoplastic disease.

Manipulation of the immune system is an important component of many prophylactic and therapeutic applications against infectious, degenerative and neoplastic diseases. The diverse repertoire of methods can be roughly divided into four approaches: active immunization (vaccination) prepares adaptive memory responses, usually prior to first exposure, and constitutes one of the most efficacious and cost-effective medical interventions. Immunization with antigens generally only leads to the induction of antibody production, whereas for instance inoculation with attenuated viruses also elicits cytotoxic effector cells [8]. In addition, active immunization can be accomplished by adoptive cell transfer. In a typical setting, dendritic cells are loaded with tumor antigens ex vivo and subsequently infused into patients to induce an adoptive immune response against cancer cells [9]. In contrast, passive immunotherapy circumvents the initial steps required for immune responses to be launched and directs the immune system efficiently to the desired medical targets. For cellular approaches, CTLs are equipped with recombinant receptors ex vivo. These cells are designed to attack neoplastic cells that express the cognate tumor-associated antigen immediately after infusion [5]. Passive immunization by administration of processed antibodies derived from human or animal donors is a well-established emergency procedure for treatment of snake-bite envenomation or post exposure prophylaxis against, for example, rabies [10]. The advantage of passive immunization is that protective antibodies can be provided in a very short time. Application of recombinant antibodies further expands the number of available targets and is increasingly important for augmentation of conventional therapies against cancer [10, 11].

Passive immunotherapies either require or can exploit modern nucleic acid-based methods, among which mRNA is the latest technology. The present review is dedicated to provide an overview on mRNA in passive immunotherapy after introducing the immunological approach as well as mRNA technology as such.

## Passive immunotherapy: general overview

### Antisera and polyclonal immune globulin preparations

**Fig. 1:**
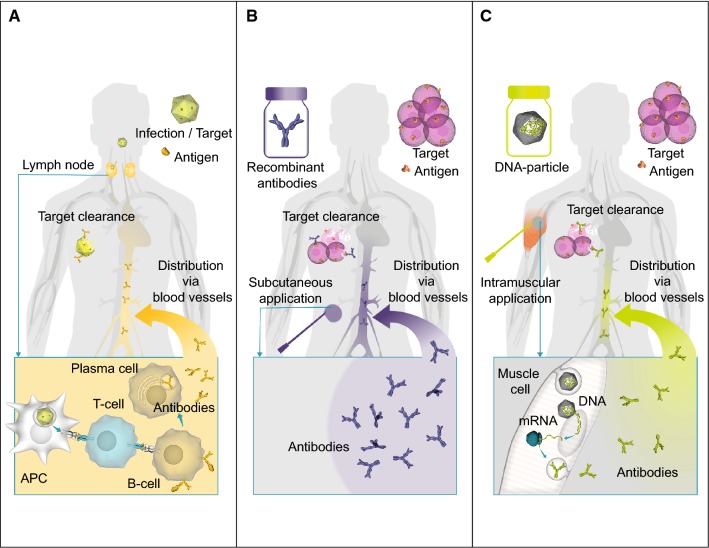
Schematic illustration comparing active immunization and passive antibody immunotherapies. **a** During active immunization triggered by natural infection or vaccination, antigenic patterns are presented by antigen-presenting cells in the lymph nodes. This leads to T cell mediated activation of antigen-specific B cells. As a consequence, B lymphocytes differentiate into plasma cells which produce and secrete antigen-specific antibodies that bind to cognate structures, finally leading to their clearance. **b** Instead of being produced by plasma B cells, antibodies can be manufactured recombinantly and administered for instance by subcutaneous injection for passive immunization. After injection, antibodies enter circulation by diffusion and act like endogenous antibodies. **c** For DNA-based passive immunization, DNA is often packaged in nanoparticles, e.g., virus capsids, which for instance can be injected intramuscularly. After uptake by muscle cells, DNA is released into the cytosol. For transcription into mRNA, the DNA has to enter the cell nucleus first. mRNA is then translated into antibodies which are secreted to bind their cognate targets

It is well known that protection raised by most of today’s licensed vaccines is primarily antibody-dependent (Fig. 1a) [8, 12]. Basically, this explains the long and successful history of passive immunotherapy (Fig. 1b). The protective capacity of serum against bacterial toxins was discovered in the early 1890s [13]. The avoidance or control of infection by such passive immunization is based on the transfer of serum and later polyclonal immune globulin (= antibody) preparations from convalescent or vaccinated humans or animals [14, 15]. Prior to the discovery of antibiotics, serum was the only antidote for bacterial diseases [16]. Thus, following successful passive immunization against diphtheria toxin [13], a whole plethora of serum or immune globulin therapies for viral and bacterial diseases as well as to neutralize snake toxins was developed [17]. Clinical benefits of serum and immune globulin therapy were demonstrated for viral diseases such as influenza, measles, and polio and bacterial infections with meningococcus or pneumococcus [18–20].

With the advent of antibiotics, the use of serum or polyclonal immune globulin preparations as antibacterial agent was largely discontinued in late 1940. However, such therapies retained a niche as treatment for venoms, toxins, and certain viral infections. In the second half of the twentieth century, polyclonal antibody preparations were developed and in part licensed for the prophylaxis and treatment of hepatitis A and B, cytomegalovirus, varicella-zoster virus, vaccinia virus, rabies, respiratory syncytial virus, West Nile virus, and various hemorrhagic fevers [21]. For instance, passive immunization against the Argentine hemorrhagic fever shows beneficial effects when applied within 1 week after the emergence of symptoms, and post-exposure treatment with human or equine immunoglobulins are recommended for rabies [22–24]. Moreover, botulism is treated with equine antitoxin [25, 26]. Such immune globulin preparations of non-human origin are particularly prone to elicit an immune response that obstructs their therapeutic use or efficacy [27]. Further disadvantages or difficulties associated with the use of serum or polyclonal globulin preparations are the often high content of non-neutralizing antibodies, batch-to-batch variations, and in case of human sources the availability of appropriate immune donors [21, 28].

### Monoclonal antibodies

In 1975, groundbreaking work described the production of monoclonal antibodies (mAb) by immortalization of B cells [29]. The resulting hybridoma technology was then rapidly exploited for clinical use, for instance, to produce a mAb to CD3 for preventing organ rejection [30]. Recombinant technologies further expanded the available therapies based on mAbs. In vitro antibody selection technologies like phage or ribosome display were developed to enable the generation of highly specific human mAbs out of libraries that may even be naïve for the specific antigens [31–36]. In the early 2000s, a high throughput technique to amplify and clone antibody genes from single human B cells was described [37, 38].

#### Full-size antibodies

**Fig. 2:**
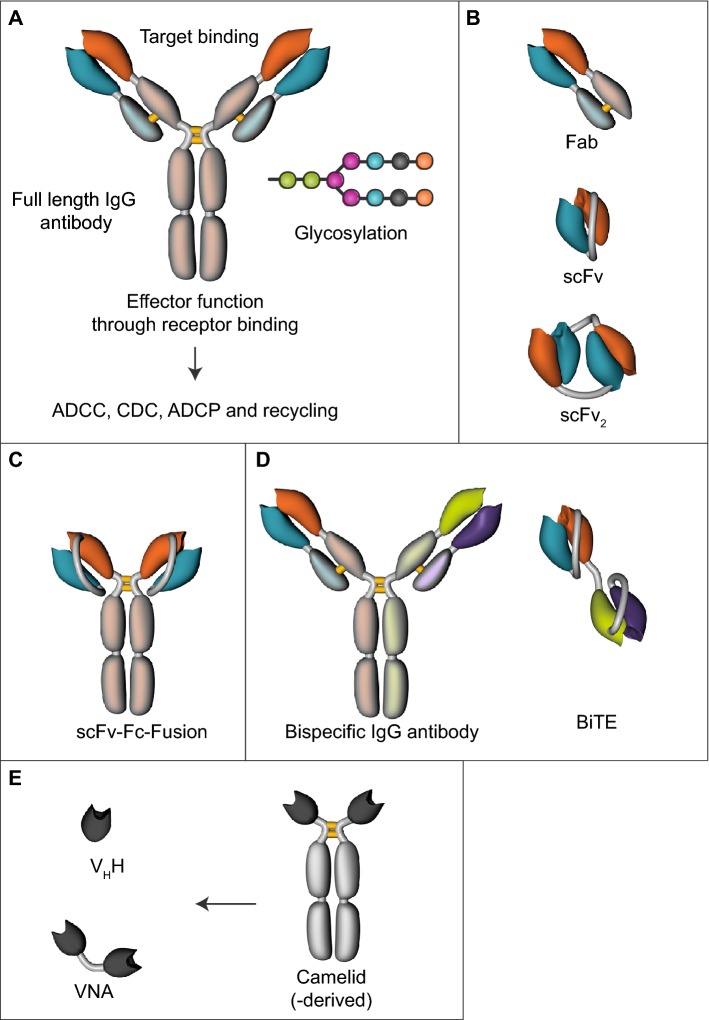
Schematic representation of antibody formats used for passive immunotherapies. **a** A full-size antibody consisting of two heavy and two light chains assembles via disulfide bonds (yellow connection). Both heavy and light chains contain constant (dim color) and variable (bright color) regions. Variable regions are involved in antigen binding. The heavy constant region contains the Fc region mediating effector functions and binding to the FcRn for antibody recycling and thus enhancing antibody serum half-life. Antibodies can be modified by glycosylation in the ER/Golgi posttranslationally. **b** Derivatives of full-size antibodies include Fab fragments and different types of scFvs. **c** To enhance the half-life or to introduce effector functions, scFv constructs can be fused to an Fc region. The resulting single-chain construct can assemble into a homo-dimer. **d** More complex antigen-binding properties can be introduced by the generation of bi-specific antibodies or covalently linked scFv domains with different binding affinities, such as bi-specific T-cell engagers (BiTE). **e** Camelids produce antibodies that lack light chains. Single domain V_H_H derived from such antibodies could be used for therapeutic purposes. More sophisticated approaches utilize fusions of two or more antigen-binding domains with identical or different antigen specificity termed V_H_H-based neutralizing agent (VNA)

Although recombinant mAb technology is exploiting a large variety of different antibody formats (Fig. 2), the prevalent type is still a full-size antibody of the IgG class. In addition to the variable domains essential for antigen binding, they contain constant domains including the Fc-region. The latter is important for antibody function and can mediate antibody-dependent cellular cytotoxicity (ADCC), complement-dependent cytotoxicity (CDC), and antibody-dependent cellular phagocytosis (ADCP) [39]. Furthermore, binding to the neonatal Fc receptor (FcRn) plays a role in controlling the antibody half-life which is 21–28 days for human IgG [40–42]. FcRn rescues bound antibody from degradation by transporting it back to the cell surface where it is released into the extracellular space [43, 44]. Specific mutations in the Fc region that increased the affinity to FcRn have been shown to prolong antibody half-life up to fivefold [45]. In addition to the impact of FcRn binding, half-life also benefits from the large size of IgGs. It obstructs antibody clearance by the kidney as well as metabolization by cytochrome P450 [42, 46]. The downside of the large size is the correspondingly low access to and penetration of tissue which can affect therapeutic efficacy [47].

Full-size antibodies are often posttranslationally glycosylated, modulating Fc function. Although aglycosylated IgGs can be produced in bacteria [48], modern production processes usually take advantage of the cellular machinery for advanced posttranslational maturation and secretion of eukaryotic cells [48–51]. The vast majority of approved therapeutic antibodies are currently produced in mammalian cells [52, 53]. Production processes have been optimized especially for the predominant production system, the continuous Chinese hamster ovary (CHO) cell line. CHO cells secrete antibodies with negligible non-human glycoforms [54] and are also amenable to glycoengineering [55]. Differences in glycoforms depend on the production system and affect distribution and stability of the antibody, Fc effector function and immunogenicity in recipients [54].

#### Antibody fragments

Since traditional expression hosts such as *E. coli* do not allow efficient production of full-size antibodies, smaller proteins consisting of fragments derived from the variable domains were developed as promising alternatives. Such single-chain variable fragments (scFv) and various derivatives thereof preserve antigen binding while facilitating manufacturing (Fig. 2b, c) [56]. Another type of antibody fragment is derived from camelids or cartilaginous fish. These animals produce single-domain antibodies devoid of light chains (Fig. 2e) [57, 58]. Since antigens are recognized by a heavy-chain-only V_H_ domain (V_H_H) in camelids [59], the variable V_H_H fragment can be easily engineered into nanobodies that offer additional advantages such as improved heat and pH stability [60]. Moreover, they can also be assembled into V_H_H-based neutralizing agents (VNAs) (Fig. 2e) [61]. Various studies demonstrated that multivalent formats were more effective than monovalent single-domain antibodies [62, 63]. Notably, all formats based on antibody fragments can be relatively efficiently produced with less expensive bacterial expression systems, typically employing *E. coli* [64, 65]. The antibody fragments produced in this system are often targeted to the oxidative environment of the periplasm using specific signal peptides to foster disulfide bond formation and proper folding [64, 65]. Moreover, enhanced expression of chaperones and cytoplasmic oxidases has been demonstrated to increase the yield of antibody fragments [48, 66].

Small antibody fragments were also the basis for developing the concept of bispecific antibodies more than 20 years ago. Initially, single chain antibodies with a different binding specificity were fused to the C-terminal ends of heavy chains of IgGs [67]. Generation of first bispecific IgG molecules benefited from the knob-into-hole technology [68]. Today, many different bispecific antibody formats combining two different antigen binding domains in one molecule are available (Fig. 2d) [69–72]. Among them, bispecific diabodies (bi-(scFv)_2_) and BiTE antibodies are prominent examples [73, 74]. In general, bispecific antibodies can be deployed to target therapeutic substances such as toxins, radionuclides, and drugs as well as effector cells like CTLs to the site of cognate antigen expression [75].

Associated with their small size, many formats using antibody fragments are cleared by renal elimination [76, 77]. Moreover, in the absence of an Fc region, recycling by the FcRn rescue mechanism cannot take place [77]. As a consequence, these formats usually reveal short plasma half-lives [77]. For instance, bi-(scFv)_2_ antibodies have a serum half-life of less than 2 h which usually requires continuous infusion [78]. In case of the BiTE blinatumomab, the antibody is usually administered daily due to its short half-life [79]. Possible strategies to extend serum half-lives are site-specific PEGylation and fusion to an Fc region [80, 81]. However, the latter approach would negate various advantages of antibody fragments including their better and faster tissue penetration [41, 82]. It has been shown that small single-domain antibodies could even cross the blood–brain barrier [83]. In case of an anti-rabies antibody, this allowed partial rescue of mice challenged with virus injection into the brain in contrast to full-size immunoglobulins [84, 85].

#### Clinical status quo

Today, monoclonal antibodies play an important role in the therapeutic armamentarium. Dozens of antibodies have been licensed to treat cancer, rheumatoid arthritis, multiple sclerosis, psoriasis, allergy, systemic lupus, and other diseases. In addition, mAbs have shown promise in protecting against various microorganisms, viruses, and fungal infections as well as in treating neurodegenerative diseases [86–89]. However, mAbs for infectious disease indications are still scarce among licensed products. Palivizumab for respiratory syncytial virus prophylaxis in high-risk infants was the first antiviral mAb approved by the FDA [90]. Since then, antibodies against anthrax and rabies in India became available. In addition, bezlotoxumab binding to *Clostridium difficile* toxin is used to prevent recurrent bacterial infections [91]. mAbs for treating cancer are still the largest group of licensed products. Here, rituximab, directed against the transmembrane protein CD20 on the surface of B lymphocytes, was the first mAb in clinical use [92]. However, as for many other mAbs in antitumor therapy, high doses are needed to obtain clinical efficacy [93]. A successful example for a bispecific antibody is the first-in-class BiTE against CD19/CD3, blinatumomab, which is approved for the treatment of acute lymphoblastic leukemia [94–96].

### Cellular immunotherapy

In addition to the administration of immunoglobulins, passive immunity can also be conferred by transferring functionalized immune cells. Adoptive transfer of CTLs was shown to be a potent therapeutic means to treat both viral infections and cancers [97–99]. To this end, T cells can be equipped with an additional T-cell receptor (TCR) or a chimeric antigen receptor (CAR) [100]. While CARs are limited to the binding of surface antigens, TCRs recognize MHC-presented peptides derived also from intracellular proteins. The first successful clinical trial with an engineered TCR on CTLs demonstrating tumor regression was reported in 2006 [99]. Subsequently, passive immunotherapies with TCR-engineered T cells became an important approach for antitumor treatments [101]. Efficient targeting and killing of cancer cells expressing the respective antigen in patients with various forms of cancer including metastatic melanoma, synovial sarcoma, and colorectal carcinoma have been demonstrated [99, 102–104]. A possible problem specific to the use of TCR-engineered T cells is the presence of an endogenous TCR. Mispairing of endogenous and introduced α- and β-chains may create new specificities with potential reactivity to host molecules [105, 106]. To avoid such mispairing, the use of γ/δ T cells has been suggested, since γ/δ-chains do not pair with α- or β-chains [107–109]. Alternatively, gene editing is now being explored to disable endogenous TCR expression [110, 111].

The concept of CARs was developed in 1989 [112] and then refined using a scFv fragment to obtain antibody-like receptor specificity without the need to transfer multiple genes [113]. First generation CARs consisted of an antigen-specific scFv, fused to a transmembrane and intracellular CD3ζ TCR signaling domain, conferring transient activation and cytotoxicity to T cells [114]. Upon target binding through the scFv domain, the engineered T cell is activated in an MHC-independent manner [115]. Subsequent generations of CARs were improved with respect to cytotoxicity and persistence by including additional co-stimulatory domains such as CD28, OX-40 or 4-1BB [114, 116–118]. T cells engineered with such CARs targeting specific tumor antigens are remarkably successful in treating hematological malignancies like leukemia and lymphoma [119, 120]. For instance, CD19-directed CAR T cells repeatedly revealed complete and durable remissions in patients with B-cell acute lymphoblastic leukemia (B-ALL) [121–123]. In contrast, CAR T cell therapies face various challenges for solid tumors [124].

At present, the most common techniques for generating TCR- or CAR-engineered T cells utilize viral gene transduction with retro- or lentiviral vectors [125]. However, permanent expression of the transgenic receptor mediated by this efficient technology can be disadvantageous in case of therapy-related severe toxicities due to accidental cross reactivity [126–128]. On-target/off-tissue and off-target toxicities by engineered T lymphocytes attacking healthy host cells as well as cytokine release syndrome are feared side-effects which were repeatedly reported when virus vector transduced cells were applied [129–132]. Another concern of using retroviral vectors is the potential to induce insertional mutagenesis and genotoxicity in effector cells [133–136]. Hence, more precise cell manipulations are currently under investigation. Among them, gene editing of primary human T cells was recently demonstrated to be an efficient approach [137].

### DNA-based antibody expression in vivo

While T cell engineering for adoptive transfer inevitably requires the use of nucleic acids encoding a target-specific receptor, antibody immunotherapy can deploy recombinant proteins. However, as pointed out above, maintaining therapeutically effective levels may require frequent administrations dependent on clearance and indication [138]. Thus, DNA-mediated antibody expression directly in the body may represent an attractive alternative to administration of recombinant proteins (Fig. 1c). Both, plasmids as well as viral vectors have been used for passive immunization. Although efficient in small animal models, strong expression of recombinant genes using unformulated DNA does not scale well to larger animals (including primates) [139]. Consequently, application of recombinant adeno-associated viruses (AAVs) is currently the preferred method for transduction of the antibody gene of interest [140]. Early work reached single digit µg/ml serum titers [141]. Later studies with advanced vector designs reported expression levels in the high µg/ml or even in the single digit mg/ml range [142–144]. Much work has been done in the field of HIV prophylaxis. Here, a single intramuscular injection of recombinant AAV was demonstrated to elicit peak antibody titers above 100 µg/ml in mice [142]. Treated mice revealed substantial anti-HIV antibody levels for more than a year and were protected from HIV-1 challenge.

Although AAV usually maintains an episomal state, this vector still harbors an inherent risk of insertional mutagenesis. In patients with hepatocellular carcinomas integration of AAV2 into known cancer genes was observed [145]. Moreover, AAV-based immunotherapy faces various issues regarding immunogenicity [146–148]. A substantial percentage of the population has already been in contact with the used virus and consequently shows pre-existing immunity limiting the efficacy of treatment [149, 150]. The induction of anti-viral responses during immunotherapy may have similar consequences if a single virus serotype is used in repeated treatments [148]. Pre-existing or induced immunity could lead to clearance of the viral vector and/or AAV-transduced cells.

Finally, gene delivery by AAV has been reported to induce immune responses against the encoded protein. Even when using an endogenous gene such as erythropoietin (EPO), some macaques receiving EPO-encoding AAV intramuscularly developed severe autoimmunity against the protein [151]. When non-human primates were treated with AAV vectors expressing antibody or antibody-like proteins, titers dropped rapidly in some animals [152]. One reason is the possibility of inducing anti-idiotype antibodies which requires immune suppression to obtain sustained expression [153]. Further studies indicated that primates may be more prone to develop a robust T cell response to the AAV-encoded protein compared to mice [154].

In summary, the risks of insertional mutagenesis and genotoxicity, long-lasting expression without control in case of adverse events, and various potential issues regarding immunogenicity associated with viral vectors that may limit efficacy emphasize the high demand for other vectors for passive immunotherapy than DNA. How mRNA offers a viable option to meet the demands is reviewed in the following sections.

## mRNA as therapeutic: technological considerations and first examples

The cellular machinery uses mRNA as a transient carrier of genetic information for the synthesis of proteins. Based on this fundamental biological concept administering exogenous mRNA represents an alternative to DNA-mediated protein delivery in vitro and in vivo. Using mRNA instead of DNA as therapeutic substance is attractive due to the absent risk of insertional mutagenesis. Moreover, efficient expression is even obtained in non-dividing cells, since mRNA does not require a nuclear phase for activity. Compared to the delivery of proteins and peptides, mRNA may prolong the availability of effector molecules, however, not as much as DNA. In contrast to the latter, mRNA therapy, therefore, has to cope with the short half-life in vivo of exogenously delivered mRNA as for instance indicated by mRNA-mediated VEGF expression in myocardium returning to baseline within 72 h [155]. While this may be a therapeutic disadvantage in various instances, it can be considered advantageous from a safety perspective, particularly in case of adverse events.

mRNA was first employed for the expression of a protein of interest in the early 1970s when RNA preparations were microinjected into *Xenopus* oocytes and synthesis of the encoded protein was demonstrated [156, 157]. In 1989, the group of Inder Verma presented a reliable method to efficiently introduce RNA into a variety of cells using a cationic lipid [158]. Almost at the same time, mRNA-mediated protein expression in vivo was demonstrated after direct injection into mouse muscle [159]. Much of the early work on a potential therapeutic use of mRNA focused on the development of active vaccination approaches, in part since low amounts of antigen suffice due to the amplifying nature of the immune response. Subcutaneous injection of liposome-encapsulated, antigen-encoding mRNA was the first example of eliciting an antigen-specific CTL response in mice [160]. Gene gun delivery of mRNA into mouse epidermis provided the first evidence of an antigen-specific antibody response [161]. In addition, mRNA turned out to be a potent means to load dendritic cells with antigens to convert them to tailored antigen-presenting cells in vitro and in vivo [162]. Later, a new vaccination protocol was introduced which elicited a complete adaptive immune response consisting of antigen-specific antibodies and T cells with lytic activity without requiring any transfection reagents, special equipment or heterologous boost [163]. From a retrospective view, this event marked the starting point of the commercial development of mRNA vaccines.

### Building blocks for therapeutic mRNA

**Fig. 3:**
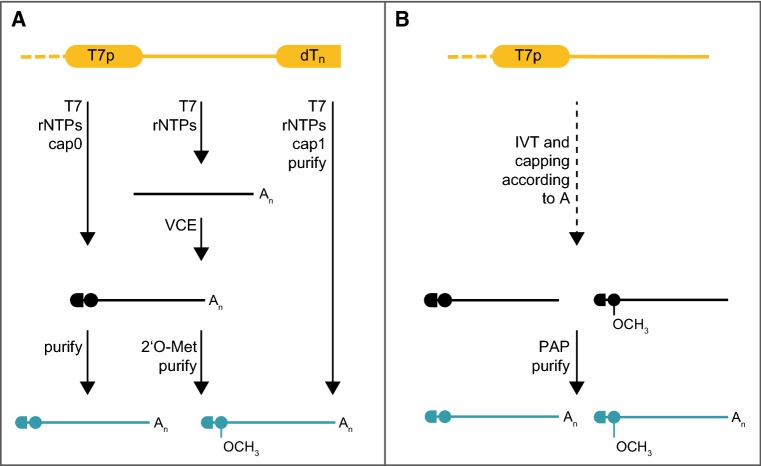
Flowchart for the manufacturing of IVT mRNA. **a** Starting with a DNA template (yellow) harboring all essential mRNA sequences including a poly(A) tail, different processes can be used to generate mRNA with either a cap0 or a cap1 structure (Fig. 4). T7p, T7 RNA polymerase promoter; dTn, poly(dT); T7, T7 RNA polymerase; rNTPs, ribonucleotides; cap0, cap1, cap analogs; VCE, Vaccinia virus capping enzyme; 2′O-Met, Vaccinia virus methyltransferase. **b** If a poly(A) tail is not encoded in the DNA template, it can be enzymatically added to the mRNA after IVT and capping by a poly(A) polymerase (PAP)

In the minimal structure, mRNA contains a protein-encoding open reading frame (ORF) flanked at the 5′- and 3′-end by two elements essential for the function of eukaryotic mRNA: the “cap”, a 7-methyl-guanosine residue (m7G) bound to the 5′-end of the RNA via a 5′–5′ triphosphate bond, and, with the exception of histone mRNAs, a poly(A) tail at the 3′-end [164–166]. Synthetic mRNA is transcribed in vitro from a plasmid DNA template that contains at least a bacteriophage promoter, the ORF, and a unique restriction site for linearization of the plasmid to ensure defined termination of transcription. Typically, the template also contains a poly(d[A/T]) sequence transcribed into poly(A). Alternatively, the poly(A) tail can be generated by enzymatic in vitro polyadenylation of transcribed RNA. The cap may be incorporated into the transcript during transcription by including an m7GpppG dinucleotide as a structural homolog of the endogenous cap structure in the transcription reaction. Different options for how to derive IVT mRNA are summarized in Fig. 3.

#### Cap structures

**Fig. 4:**
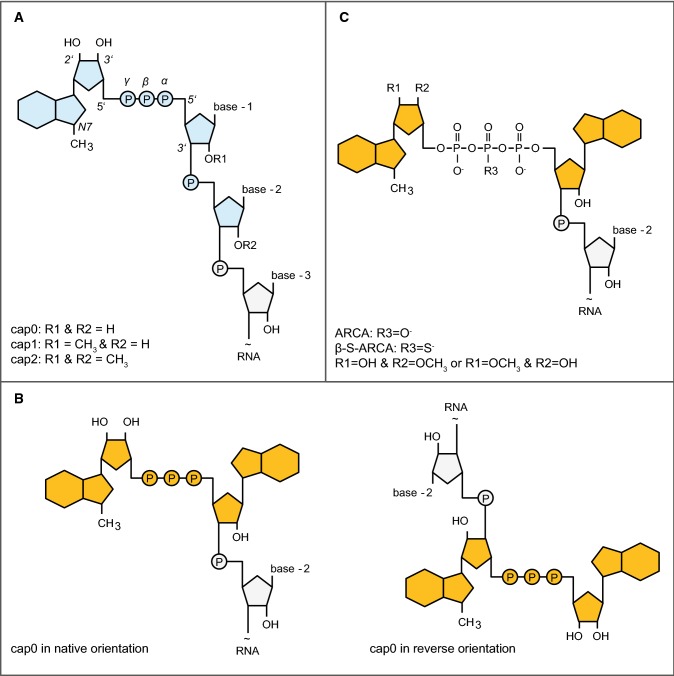
Schematic representation of different cap structures. **a** The typical 5′ cap of eukaryotic mRNAs. A guanosine is methylated at position 7 and linked to the first nucleotide of the mRNA by an unusual 5′ to 5′ triphosphate bridge. Depending on the degree of methylation of the first two bases of the mRNA, the full 5′ terminal structure is referred to as cap0, cap1 or cap2. The CleanCap™ analog, a trinucleotide introducing a cap1 structure during IVT, is indicated in blue. **b** A plain cap0 analog (orange) is incorporated in two orientations during IVT. **c** Inverse orientation can be avoided using anti-reverse cap analogs (ARCAs, highlighted in orange). Such analogs are characterized by the presence of a methoxy group at either C2′ or C3′ of m^7^G. To improve resistance to decapping, a phosphorothioate was positioned in the 5′–5′ bridge of ARCA (β-S-ARCA)

Various elements of an mRNA molecule contribute to the level and duration of expression of the encoded protein. The cap structure is required for efficient translation and stabilizes mRNA towards exonucleolytic decay [167–169]. Various structures have been repeatedly used for IVT mRNA (Fig. 4). The basic m7GpppG cap analog is incorporated in both orientations into the RNA by the bacteriophage RNA polymerase [170]. However, reverse incorporation of the cap analog results in mRNA molecules that lack the m7 methylation at the cap and are not recognized by the translational machinery [171]. Substitution of the hydroxyl group in C2′ or C3′ position of the m7G with a methoxy group prevents reverse incorporation of the cap analog by inhibiting elongation at the m7G. The dinucleotide is, therefore, called ‘anti-reverse cap analog’ or ARCA [171, 172]. ARCA-capped mRNA revealed increased as well as prolonged protein expression in cultured cells and enhanced reporter protein expression in mouse dendritic cells up to 20-fold [173, 174]. For further optimization of the cap structure, modifications were introduced within the triphosphate linkage to inhibit decapping. Substitution of a non-bridging oxygen in the β-phosphate moiety of an ARCA by sulfur results in the β-S-ARCA dinucleotide. While β-S-ARCA maintains recognition by the translational machinery, protein expression from mRNA capped with β-S-ARCA was extended in HC11 cells and immature DCs, but not in mature DCs [175, 176].

Enzymatic capping represents an alternative to the co-transcriptional approach, avoiding a N7-unmethylated cap as does ARCA. To this end, mRNA is transcribed without cap analog and subsequently capped using the vaccinia virus capping complex [177]. This complex with triphosphatase, guanylyltransferase and (guanine-7)-methyltransferase activity adds a natural cap to the 5′-triphosphate of an RNA molecule. Such a cap0 structure can be further converted into a cap1 group by O-methylation of the ribose of the cap-proximal nucleotide using the vaccinia virus 2′O-methyltransferase [178]. Cap1 (and cap2 harboring a further O-methyl-ribose at the subsequent nucleotide) structures are typical of eukaryotic mRNA and are recognized less by cytosolic RNA sensors of the innate immune system, thereby rendering the mRNA less immunostimulatory. For instance, RIG-I is activated by cap0 but not cap1 mRNA and IFIT1 binds cap0 mRNA more tightly than cap1 molecules [179, 180]. Such sensor-mediated immune stimulation may in turn negatively impact mRNA translation [181]. It is interesting to note that since recently a new cap analog, CleanCap, is available which enables the cotranscriptional incorporation of a cap1 structure into IVT mRNA.

#### Sequences

Like the cap structure at the 5′-end, details of the poly(A) tail at the 3′-end influence translation and stability of mRNA [167, 182]. While numerous studies demonstrated a positive effect of a poly(A) tail and some correlation of effectiveness and length of the element, details of observations were remarkably variable. The majority of studies indicate an enhancement of translation when extending the poly(A) length from approximately 60–70 to 100–150 nucleotides or by tail extension using enzymatic polyadenylation [174, 183–185]. However, effects were mostly moderate, but reached a maximum of a 35-fold increase in one particular setting. In contrast, another study reported an optimum of approximately 60 nucleotides; protein expression declined with further increasing poly(A) length [186]. In addition to poly(A) length, one report suggests a positive role of using a type IIS restriction enzyme for DNA template linearization to obtain a free poly(A) end rather than one extended with unrelated nucleotides [183].

Further elements that can affect mRNA translation and/or half-life are untranslated region (UTR) sequences flanking the ORF sequence. Trans-acting regulatory RNA-binding proteins (RBPs) interact with distinct RNA sequence elements, thereby affecting ribosome recruitment and transit [187]. For instance, β globin 5′- and 3′-UTRs, duplicating the β globin 3′-UTR, the 5′-UTR of tobacco etch virus, and a structure of the 5′-UTR of human heat shock protein 70 all enhanced mRNA translation in mammalian cells [158, 183, 188, 189]. According to a very recent survey of a combinatorial UTR library, 5′-UTR sequences appear to be most critical for protein expression [190]. In contrast, 3′-UTRs seem to be the key driver for mRNA half-life as exemplified by the stabilizing effects of the α globin 3′-UTR as well as a duplication of the β globin 3′-UTR [183, 191].

Codon usage of the ORF sequence also affects translation efficacy in many species. Although in humans codon usage bias does not correlate with tRNA levels and gene expression [192, 193], still increased protein expression from mRNA upon codon usage optimization has been reported. For instance, codon usage adaptation of HIV-1 gag improved protein yield from mRNA approximately 1.6-fold in a human T lymphocyte cell line [194]. A more pronounced increase in protein expression as a result of codon optimization was reported upon transfection of mRNA encoding angiotensin-converting enzyme 2 into A549 and HepG2 cells [195]. However, alternatively to a direct effect of codon usage, the enhancement may be an indirect result of the use of modified nucleotides, the content of which was altered by codon optimization. Furthermore, coding sequence engineering exploiting more advanced concepts like codon optimality may prove valuable in designing therapeutic mRNAs of high efficacy. According to recent insights, codon usage may also affect fidelity of translation or the stability of transcripts [196].

In addition, ORF as well as UTR sequences can have an effect on immunostimulation and thus on translational activity. Initially, researchers applied mRNA containing only the four unmodified bases A, U, C, and G [158–160, 197]. In this context, for instance, U-rich sequences, as well as several RNA structural features were described as immunostimulatory due to interactions with various RNA sensors such as Toll-like receptors (TLR), RIG-I, and protein kinase R (PKR) [198–205]. As a consequence, development of mRNA therapies was hampered by the immunogenicity of in vitro transcribed mRNA.

#### Immunostimulation

The consideration of mRNA for therapeutic purposes gained momentum by the finding that incorporation of modified bases into in vitro transcribed mRNA reduced immunostimulation of such preparations. Various modified bases found in natural RNAs suppressed recognition by TLRs in vitro [206]. Among these, particularly pseudouridine increased translation and stability of mRNA [207]. In addition to the effect on TLR binding, replacement of uridine by pseudouridine affected binding to and activation of further sensors such as PKR and 2′-5′-oligoadenylate synthetase, contributing to higher and longer protein expression [208, 209]. However, the effects of mRNA modifications on translation, immunostimulation and resulting protein expression appear to be variable, for instance dependent on the type of target cells. In vitro testing of various modifications and combinations thereof revealed decreased protein yield for any of them in a context dependent manner, while most of them reduced immunostimulation in RAW124.7 macrophages [210].

As found in vitro, pseudouridine modification of mRNAs reduced immunostimulation and increased level and duration of protein expression after intravenous (IV) or intraperitoneal administration of formulated mRNA in mice [188, 207]. In contrast, another study on systemic administration of nanoparticle-complexed mRNA concluded that neither immunostimulation nor protein expression benefited from pseudouridine modification [211]. After intradermal or intramuscular injection of formulated mRNA, only *N*1-methyl-pseudouridine, but not pseudouridine, substantially enhanced expression [212]. In line, a different study applying lipid nanoparticle (LNP)-formulated mRNA intradermally found that replacement of uridine with *N*1-methyl-pseudouridine resulted in much increased and longer lasting protein expression [213]. Notably, recent studies revealed that also endogenous eukaryotic mRNA harbors various modified nucleotides [214–217]. However, the total level of modification is rather low which is in strong contrast to the usually 100% replacement of an unmodified nucleotide in IVT mRNA. Moreover, heavy nucleotide modification appears to interfere with the function of translation-enhancing RNA elements such as UTRs and internal ribosomal entry sites (IRESes) [218].

**Fig. 5:**
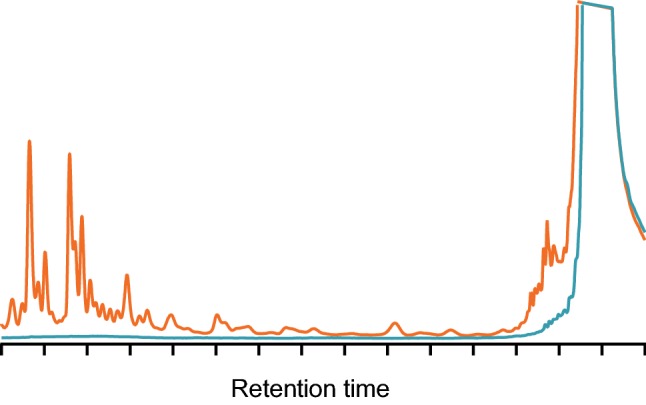
Prototypical analytical HPLC profiles demonstrating the effect of chromatographic purification of IVT mRNA. IVT mRNA preparations may be contaminated with smaller or larger by-products such as abortive transcripts or transcripts from traces of non-linearized DNA template. Analyzing a raw IVT mRNA preparation by HPLC, the various erroneous transcripts contained are apparent in the chromatogram (orange). After separating the various transcripts by size on a preparative HPLC column and isolating the mRNA of interest, reanalysis of the purified mRNA demonstrates complete removal of all contaminants (blue)

At the time when modified nucleotides were introduced to minimize immunostimulation of IVT mRNA, the importance of stringent purification of such preparations was recognized. Chromatographic purification, particularly HPLC, can separate mRNA according to size, thereby removing smaller or larger by-products such as abortive transcripts, mRNA from traces of non-linearized DNA template or double-stranded RNA (Fig. 5) [219, 220]. Such purification enhanced protein yield, most probably by enriching functional transcripts and depleting contaminants causing detrimental immunostimulation [220, 221]. The latter is corroborated by the finding that stringent purification reduced the beneficial effect of chemical modification or even made it dispensable, particularly in combination with specific sequence-engineering of the mRNA [218, 220]. Importantly, the specifics of mRNA formulation are another layer that can influence activation of the innate immune system by masking mRNA from recognition by sensors, particularly TLRs. For example, immune responses after mRNA administration into the central nervous system were effectively suppressed by the use of a nanomicelle formulation compared to naked mRNA [222].

### mRNA expression of therapeutic proteins in vivo

After in vivo administration of mRNA was proven to be feasible, the concept of using mRNA as a basis for therapeutics was pursued almost immediately. The very first report on a therapeutic effect with exogenous mRNA was already published in 1992 and described a temporary reversion of diabetes insipidus in rats by intrahypothalamic injection of vasopressin mRNA [197]. Thereafter, it took almost two decades until further studies started to demonstrate the broad potential of mRNA-based protein therapies. Meanwhile, there is a plethora of publications on a huge variety of indications comprising anemia [188, 218], hemophilia [223, 224], myocardial infarction [155, 225], cancer [226, 227], lung disease such as surfactant B deficiency and asthma [228–230], metabolic disorders [231–235], fibrosis [195], skeletal degeneration [236], tendon impairment [237], and neurological disorders such as sensory nerve dysfunction, Friedreich’s ataxia and Alzheimer’s disease [238–240]. Whereas evidence for the therapeutic potential of mRNA is mostly restricted to mouse models, first data in swine indicate that mRNA-based protein therapies are feasible also in large animals [218, 225]. In view of the various indications, it is hardly surprising that this diversity goes along with different routes of administration and various formulations. Only very few studies looking at local administration used uncomplexed and thus unprotected mRNA [225, 228, 230, 237]. The majority of investigations built on lipid-based formulations with a clear tendency to the application of LNPs [223, 224, 231–233, 239]. Most if not all groups purified their IVT mRNA before in vivo administration. While some simply precipitated the mRNA [195, 227, 236], most used commercial purification kits. Only a few researchers applied HPLC purification [188, 218, 232]. With respect to the mRNA, the vast majority of studies used long poly(A) tails of at least 100 nucleotides. Likewise, there is a clear prevalence to chemically modified mRNA, although various examples suggest that this is not mandatory [218, 223, 239, 240]. While most mRNAs harbored 5-methyl-cytosine and/or pseudouridine initially [155, 188, 226], there appears to be a trend towards the use of *N*1-methyl-pseudouridine at present [224, 225]. Regarding the cap structure, almost all early studies cotranscriptionally generated cap0 mRNAs using ARCA [226, 228, 229]. However, since about 2 years, research groups prefer to apply mRNAs with a cap1 5′-end [223, 227, 231].

## mRNA in passive immunotherapy

### Passive cellular immunotherapy with mRNA

#### In vitro characterization

Initial attempts to apply mRNA to passive immunotherapy focused on cellular approaches for various reasons. Adoptive transfer of CTLs equipped with either an additional TCR or a CAR had shown great promise in cancers and viral infections. In contrast to typical scenarios of antibody therapy, receptor expression usually requires much lower protein levels. Furthermore, T cells are loaded with receptor-encoding nucleic acid (DNA or mRNA) ex vivo. Hence, passive cellular immunotherapy does not require sophisticated and highly efficient formulations for in vivo delivery but can build on the armamentarium of cell transfection methods. Previous work on active cellular vaccination with antigen-presenting cells that had been transfected with antigen-encoding mRNA revealed electroporation as easy and efficient means to load cells [241]. Comparison to passive cell pulsing and lipofection demonstrated that electroporation was also most efficient for transfection of T lymphocytes [242]. RNA electroporation had up to 90% efficiency without eliciting any critical toxicity [243]. Onset of transgene expression was very rapid and lasted about 7 days [243]. Receptor transfer into T cells by mRNA electroporation has now been well established for many years [243, 244]. Moreover, GMP-compliant protocols for manufacturing receptor-expressing T cell preparations via mRNA electroporation are available today [245, 246].

Electroporation of human T lymphocytes with various antigen-specific TCRs redirected them to recognize cancer cells in an MHC-dependent manner in vitro [243]. mRNA-mediated TCR expression conferred in vitro cytotoxicity to T cells for at least 72 h [244]. The lytic efficacy of such cells was comparable to retrovirally transduced lymphocytes [244, 247]. Likewise, transfection of CAR-encoding mRNAs generated cells that were lytically active in vitro. Using an optimized IVT mRNA for a CD19-specific CAR, surface expression and cytotoxic function were detectable for up to 10 days [248]. To avoid as many manipulation steps as possible in generating T cells for adoptive transfer, it was demonstrated that human peripheral blood lymphocytes instead of purified T lymphocytes could be used as well to elicit strong cytotoxicity in vitro upon electroporation of a CAR mRNA [249]. Currently, most CAR approaches deploy αβ T cells. However, γδ T lymphocytes are an attractive target as well due to their antitumor effector function which is not MHC restricted and does not require co-stimulation. Accordingly, mRNA-mediated TCR and CAR expression in such cells was investigated very recently and shown to kill target cells in an antigen-specific manner [246].

To the best of our knowledge, there is so far just one study that started to systematically analyze the role of different mRNA elements for receptor expression in T cells. To this end, the group of Carl June built on previous findings in dendritic cells which revealed the superiority of a duplicated β globin 3′-UTR over a single copy of the same element and of a long (120 nt) over a short (16–51 nt) poly(A) tail [183]. With respect to receptor expression, 150 As enhanced expression compared to 64 As [184]. A tandem repeat of the β globin 3′-UTR had also a beneficial effect, particularly in combination with a long poly(A). In contrast, the VEGF translational enhancer as 5′-UTR element had even detrimental consequences. The authors speculated that this may be due to reduced capping efficacy but did not provide data corroborating their hypothesis. However, they demonstrated the important role of the cap structure. Co-transcriptional cap0 using ARCA and an enzymatically generated cap1 structure were equivalent and outperformed the basic cap analog as well as an enzymatic cap0. Besides expression level, capping also appeared to have an effect on the persistence of expression. Modification of the ORF sequence by removing all internal ORFs had no effect on receptor production.

#### In vivo findings

T lymphocytes transfected with TCR- or CAR-encoding mRNA proved to be functional also in vivo. Robust antitumor effects were observed in various preclinical models [184, 249]. They were mRNA-specific, since mock-transfected T cells had no or very little unspecific impact. Although mRNA-mediated receptor expression is transient, a single injection of CAR T cells against CD19 was sufficient to prolong survival of mice [248]. Using peripheral blood lymphocytes instead of purified T cells for CAR mRNA transfection (see above) also enabled a strong antitumor response in vivo although those cells could not persist long-term in vitro [249]. Very recently it was shown that mRNA cannot only be used to drive receptor expression, but can support the generation of T cells for adoptive immunotherapy. By expressing a chimeric membrane protein against CD3, cells could be efficiently stimulated and expanded in vitro [250]. After transfection with an mRNA for an anti-CD19 BiTE, those cells mediated sustained reduction in tumor burden upon intraperitoneal injection.

Based on encouraging preclinical data, adoptive T cell therapies using mRNA were already subjected to first clinical testing. In a phase 1 trial on solid tumors addressing the safety and feasibility of using such cells, CAR transfected lymphocytes migrated to tumor sites after IV injection [251]. In addition, the study appeared to provide initial evidence of antitumor activity. Due to the transient nature of mRNA expression, subjects received repeated infusions of T cells. This led to an anaphylactic response in one patient who developed antibodies specific to the scFv domain of the CAR [252]. However, this could be a consequence of the murine origin of this domain. In another trial, mRNA-transfected CAR T cells were injected intratumorally in metastatic breast cancer patients [253]. Treatment was well tolerated and elicited an inflammatory response within tumors.

As discussed above, the use of viral vectors for adoptive T cell therapy has potential safety issues. In various studies, authors ascribed toxicities particularly to the persistence of receptor-expressing T cells. Regarding such concerns, mRNA-mediated TCR or CAR expression offers at least two advantages. First, mRNA does not integrate into a cell’s genome, thus excluding genotoxicity. Second, due to its transient nature, any potential toxicities accompanying treatment are temporary as well [254]. However, the increased level of safety has a substantial drawback. Apparently, clinical efficacy correlates with long-term persistence of receptor-engineered T cells [255, 256]. As a consequence, mRNA-transfected cells are expected to have limited anti-tumor activity because of rapidly declining receptor expression. Substantial mRNA translation only lasts about 1 day which translates into efficacious receptor levels on the cell surface for several days [183, 184]. Importantly, clinical studies demonstrated that IV infused T lymphocytes reached tumor sites only 2–3 days after administration [257, 258]. As a consequence, intratumoral or intraarterial administration was suggested to counteract the delayed cell arrival. The problem of transience of mRNA is further enhanced by the well-known ligand-induced receptor internalization. Upon target recognition, TCRs as well as CARs are rapidly internalized, a mechanism which is important for proper signal transduction [259, 260]. This explains why lentiviral vectors generated a more robust treatment effect than mRNA [184]. mRNA transfection can give rise to high receptor expression, equaling lentiviral vectors [261]. However, mRNA was only similarly effective during the first hours after electroporation. Later, contact to target cells strongly down-regulated receptor on the cell surface while lentiviral expression remained constant [261]. In comparison to a single transfer of cells with retroviral CAR expression, an mRNA-encoded receptor required three consecutive lymphocyte infusions to obtain a comparable antitumor effect [262]. These observations and considerations may be put into perspective at least in part by the finding that transferred T cells can become tolerized rather rapidly, thereby losing their ability to function in the tumor microenvironment [263]. Thus, frequent injection of T cells may be desired even for viral vector transduced T cells. Notably, mRNA was also considered to be of use in settings that require long-term expression for therapeutic efficacy and preclude repeated cell administration. The greater safety of mRNA-transfected T cells may make the initial testing of novel antigens and receptors with unknown on-target/off-tissue toxicity less hazardous [248].

### Antibody therapy with mRNA

Passive immunization with antibodies often requires considerable amounts of polypeptide to obtain therapeutically active concentrations after systemic administration. This poses a substantial challenge to the broad applicability of any nucleic acid mediated passive immunization strategy. Thus, compared to the very first attempts regarding in vivo protein expression with mRNA in the 1990s, the optimization of IVT mRNA to enhance and extend expression is a prerequisite for many mRNA-based passive immunotherapies. As reviewed above, great progress towards this goal was made during the last almost three decades. Another potential hurdle for passive mRNA immunization is related to delivery. To obtain high levels of in vivo antibody expression, the mRNA should be targeted to as many “producer” cells as possible which in turn should be transfected with high efficiency. To this end, the mRNA which is prone to degradation by RNases should also be protected against these ubiquitous nucleases in an appropriate manner. Moreover, a viable mRNA complexation reagent needs to be well tolerated. Notably, various commercial transfection reagents can be used to formulate mRNA and suffice research purposes. Among them, TransIT has been repeatedly used for in vivo studies [188, 218, 264]. With respect to potential therapeutic applications, the class of lipid nanoparticles (LNPs) became the most widely deployed means of complexation [223, 224, 231–233]. After IV delivery, LNPs mainly route to the liver on the basis of an apolipoprotein E (ApoE)-dependent mechanism [265]. However, such nanoparticles were demonstrated to be also applicable to intramuscular and subcutaneous administration [266].

**Fig. 6:**
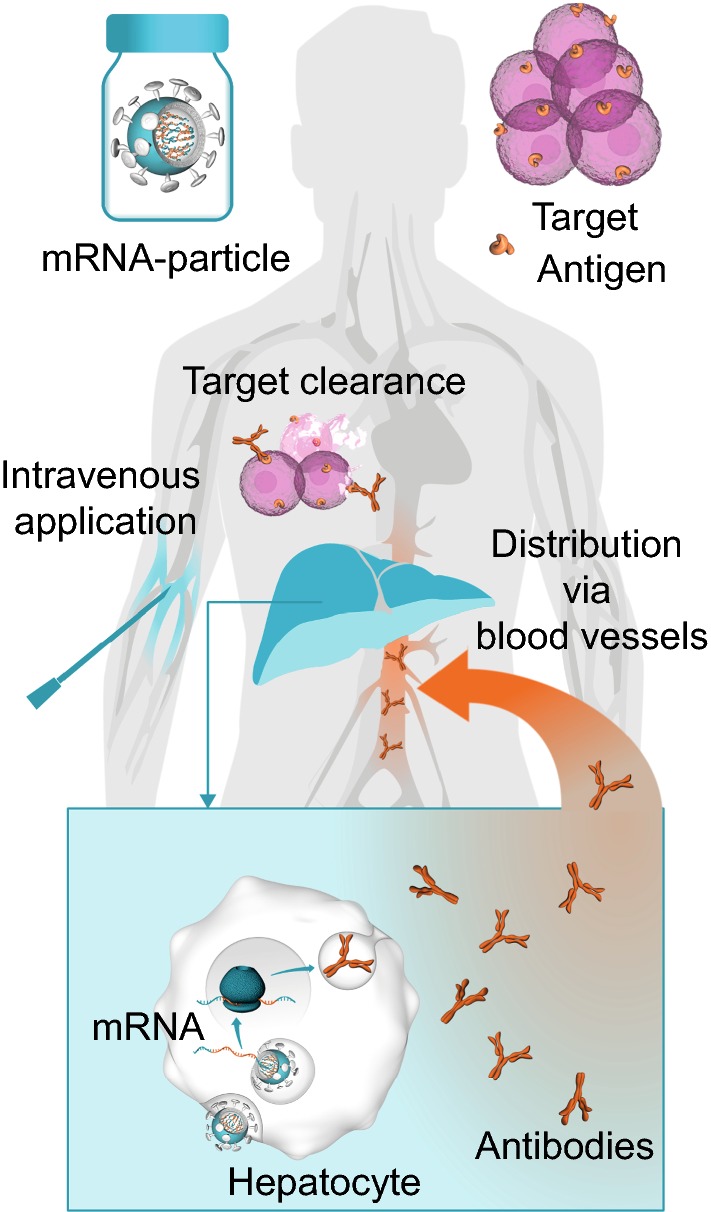
Schematic illustration of mRNA-mediated passive antibody immunotherapy. For in vivo administration, mRNA is usually formulated in nanoparticles which for instance can be administered by IV injection. For many formulations, liver is the main target organ. Upon uptake of nanoparticles by hepatocytes and release of the mRNA into the cytosol, it is translated into antibodies that are typically secreted into circulation and finally bind their cognate antigens

Advances in mRNA and formulation technology led to a couple of recent intriguing studies as to passive immunization with mRNA (Fig. 6). While the group of Drew Weissman described successful passive mRNA immunization for prophylaxis of viral infections [267], Stadler et al. demonstrated the applicability of mRNA-mediated antibody expression for cancer immunotherapy [264]. The feasibility of using mRNA for such indications was confirmed by Thran et al. who applied different mRNA-encoded antibody formats to diverse biological threats, viruses, toxins, and tumors [268]. Finally, Sabnis and colleagues presented first antibody expression data in non-human primates (NHPs) in a publication dealing with the development of novel LNP formulations [269].

#### mRNA design and formulation

Fundamental designs of antibody-encoding mRNAs reveal only a few common features but several differences (Table 1). Among the latter, the exclusive use of chemically unmodified nucleotides by Thran et al. as in a previous publication from the same group [218] may be the most prominent one, since it contrasts to all other reports. Other differences are much more diverse among these studies. Hence, they do not provide an unequivocal guidance for future work, but at least commonalities may be taken as a recommendation. Although obviously not mandatory, mRNA with cap1 structure was clearly preferred. In addition, all mRNAs harbored a poly(A) tail. The use of bipartite poly(A) elements by some groups may be owed to the experience that maintenance of long poly(d[A/T]) vector sequences is challenging and strongly dependent on bacterial strains [186]. Beyond these common RNA elements, the publications suggest that mRNA should be subjected to further optimizations to exploit its full potential for antibody expression. However, different strategies appear to be applicable, but little is known about the interchangeability of individual elements. Finally, chromatographic purification of IVT mRNA appears to be generally recommended as well (Table 1). Whether Pardi et al. actually used FPLC as stated throughout their report instead of HPLC applied by other groups is not fully clear, since they referred to earlier publications describing the use of HPLC [220, 270].

**Table 1:** Overview of the current literature on mRNA-encoded antibodies

Publication	Pardi et al., 2017 [267]	Stadler et al., 2017 [264]	Thran et al., 2017 [268]	Sabnis et al., 2018 [269]
Capping (structure)	Enzymatic (Cap1)	Enzymatic (Cap1)	Co-transcriptional (Cap0); Enzymatic (Cap1)	Not disclosed (Cap1)
5′ UTR	Tobacco etch virus	Tobacco etch virus	Hydroxysteroid (17-β) dehydrogenase 4	Unknown origin
3′ UTR	Not disclosed	F-I (not yet characterized)	Human albumin	Human alpha globin
Polyadenylation (structure)	Vector-encoded (monopartite; approximately A100)	Vector-encoded (bipartite; A30-linker-A70)	Enzymatic adenylation (bipartite; A64-linker-PolyA)	Poly(A) tail (details not disclosed)
Base modifications	*N*1-methyl-pseudouridine	*N*1-methyl-pseudouridine	Unmodified	*N*1-methyl-pseudouridine
Codon usage	Not disclosed	Sequence provided	GC enrichment (disclosed in [218])	Not disclosed
mRNA purification	FPLC	HPLC	HPLC	Not disclosed
Formulation/delivery	LNP/intravenous	TransIT/intravenous	LNP/intravenous	LNP/intravenous
Dose (range)	1–1.4 mg/kg	Approx. 0.25 mg/kg^a^	0.06–2 mg/kg	0.3 mg/kg
Species	*M. musculus*	*M. musculus*	*M. musculus*	*M. fascicularis*
Maximum titer across experiments (range)	70–200 µg/ml	7 µg/ml	20–400 µg/ml	4 µg/ml

^a^Due to lack of information, the value was calculated here for mice of 20 g in weight

Pardi et al. encoded a well-known, broadly neutralizing antibody against HIV-1, VRC01 [267]. To this end, heavy and light chains of the full IgG antibody were represented on separate mRNA molecules. For delivery, heavy and light chain mRNAs were mixed in a molar ratio of 1:1. Likewise, Thran et al. used separate mRNAs to encode heavy and light chain of various full IgG antibodies [268]. Titration of heavy and light chain mRNA found a molar ratio of approximately 1.5:1 to be optimal for co-delivery. Neither report provides a rationale for encoding chains on separate molecules. In principle, a bicistronic construct separating heavy and light chain by an IRES sequence or an mRNA for a polypeptide where a 2A sequence between heavy and light chain would lead to separate antibody chains by ribosome skipping could have been used [271]. For Pardi et al. the observation that modified nucleotides can hamper the function of IRES elements may have affected the selection [218]. Sabnis et al. also worked with a full IgG antibody, directed against influenza A, but did not provide any details on how heavy and light chain were represented [269]. In contrast, Stadler et al. chose BiTE antibodies directed against TCR-associated CD3 and one of three different tumor-associated antigens (TAAs) [264]. They displayed the BiTEs as Fab(scFv)_2_ or scFv_2_ molecules but focused on the latter format. Their findings on single-chain antibodies are complemented by Thran et al. whose work covers single domain-derived VNAs in addition to IgG antibodies [268]. For IV administration of antibody-encoding mRNA all but the group of Ugur Sahin used LNP formulations which, however, may differ from each other in composition (Table 1). The latter team exploited TransIT but switched the route of administration which had been intraperitoneal in previous studies [188, 218]. As with LNPs, nanoparticles were shown to mainly target the liver upon IV injection [264].

#### mRNA-mediated antibody expression

Drew Weissman’s group administered 30 µg of VRC01-encoding mRNA in most in vivo studies [267]. This corresponded to doses between 1 and 1.4 mg/kg due to differences in mouse weight among experiments. Antibody serum titers 24 h after administration, the earliest time of analysis, ranged between approximately 80 and 200 µg/ml in various mouse strains. Obviously, slight differences in dosage, as well as the respective strain contributed to varying peak levels. Notably, increasing the administered mRNA dose in steps of two enhanced serum titers by more than twofold with each step. Moreover, 30 µg of mRNA in LNPs generated higher serum titers than 600 µg of recombinant VRC01 protein. The kinetics of antibody serum titers revealed an accelerated decline after about a week in BALB/c mice. The kinetics in NSG mice appeared to be basically the same, since the level at 1 week after single administration was largely the same as in BALB/c at this time. The observed kinetics may also explain why weekly injections of mRNA-LNPs in NSG mice did not show additive effects on serum titers at the times of analyses. Since measurements were conducted 7 days after each treatment, antibodies from the preceding injection probably dropped to background levels within this 2-week period as observed in BALB/c animals. Such accelerated decline of protein titers after a few days is often indicative of the induction of an anti-drug antibody (ADA) response [139]. The likelihood of such a response may be particularly high for the reported experiments, since the authors expressed a human antibody in mice. The emergence of ADAs cannot be fully ruled out because animals were not analyzed accordingly. However, the apparently similar kinetics in immunocompromised NSG mice which are unable to develop ADAs suggests a different explanation for the pharmacokinetics. Possibly, the mRNA continues to express antibody for a few days which would inevitably lead to a seemingly extended antibody serum half-life during that period. Only after expression ceases, the actual shorter antibody half-life becomes evident. While this could also easily explain the serum profile of repeated treatment of NSG mice, it remains hypothetical due to the lack of respective analyses.

Stadler et al. first characterized their mRNA in vitro demonstrating expression and secretion of functional antibodies [264]. In a PBMC-mediated killing assay, mRNA-derived BiTE antibodies targeted CTLs to tumor cells via binding to CD3 on PBMCs and to the cognate TAA on tumor cells, thereby inducing T cell activation and tumor cell lysis. These antibodies were equally potent as the corresponding recombinant protein. In immunodeficient NSG mice, antibody plasma levels peaked within 6 h, but rapidly declined by more than 50% within the next 18 h. Subsequently, the decrease of BiTE titers became much slower. The authors did not provide an explanation of this striking kinetics. A pharmacokinetic analysis in non-tumor-bearing mice could have elucidated whether the initial kinetics reflects the trapping of antibody in the engrafted tumor until saturation of binding sites. BiTE plasma levels above background for a few days were in accordance with the sustained ex vivo cytotoxicity of plasma from mRNA-treated mice. In contrast to the antibody plasma kinetics, cytotoxicity showed a steady and slow decline during the observation period. 0.05 µg of mRNA were already sufficient to obtain strong plasma activity in the ex vivo killing assay. 5 µg of mRNA (approx. 0.25 mg/kg) were comparable to 4–7 µg of recombinant antibody with respect to peak plasma concentrations that were in the range of 6.5 µg/ml in NSG mice. As opposed to this modified and HPLC-purified mRNA, antibody plasma levels were almost undetectable with mRNA preparations without modification and chromatographic purification. Plasma titers declined much faster for recombinant protein compared to mRNA, thereby demonstrating the substantial impact of mRNA on BiTE pharmacokinetics. Consequently, only mRNA was able to maintain a sustained cytotoxic activity of plasma by weekly administrations.

The various mRNA-encoded antibodies of Thran et al. included VRC01 which had been used in Drew Weissman’s work [267, 268]. However, analyses were limited to in vitro characterization, preventing a direct comparison between studies. As observed for BiTEs, IgG and VNA antibodies produced from mRNA in vitro revealed potencies comparable to that of the respective recombinant proteins. IV administration of 40 µg of unmodified mRNA (approx. 2 mg/kg) gave rise to antibody serum titers between 15 and 400 µg/ml in immunocompetent mice. This contrasts strongly with the finding of Stadler et al. who found unmodified mRNA to be basically inactive. Differences in purification and mRNA design may be responsible for this striking discrepancy. Similar to the Weissman work, Thran and colleagues observed a disproportionate increase of antibody serum titers with elevated mRNA doses. Onset of antibody expression was rather rapid, being already substantial 2 h after injection and reaching peak levels after approximately 4 h. This confirms findings on other mRNA-mediated protein therapies showing that mRNA starts accumulating in hepatocytes within minutes after administration and leads to substantial protein levels within a couple of hours [223, 231]. Serum half-life of IgG antibodies appeared to be in the range of 1 week and thus slightly longer compared to Pardi et al. [267]. As in the latter study, one of two IgGs showed an accelerated decline after about 1 week, however, only in approximately half of the animals. Here, the expedited clearance could be assigned to the development of an ADA response against the mRNA-encoded antibody. Importantly, this response was antibody-dependent and not intimately linked with the use of mRNA. As expected, VNAs revealed a much shorter serum half-life of about 1–2 days. Compared to published kinetics data on recombinant VNAs, mRNA appeared to contribute to extended antibody availability during the first days after administration as it has been observed for BiTE-expressing mRNA by Ugur Sahin’s group. However, the lack of a head-to-head comparison hampers a detailed analysis.

While previous in vivo studies on mRNA-mediated antibody expression were limited to mice, Sabnis et al. presented expression results in NHPs using a proprietary LNP formulation [269]. A 0.3 mg/kg dose of mRNA gave rise to antibody serum titers of about 4 µg/ml 24 h after IV administration which is at least at the lower end of the range of efficacy observed in mouse studies. However, data on a different protein suggest that efficacy of the formulation may be slightly lower in NHP than in mouse. Whereas a 0.5 mg/kg dose induced protein levels of approximately 7 µg/ml in mice, a 0.2 mg/kg dose generated protein titers between 200 and 800 ng/ml in NHPs.

#### In vivo efficacy of mRNA-encoded antibodies

All mouse studies on mRNA-encoded antibodies investigated their therapeutic efficacy. Pardi et al. used two different humanized mouse models to demonstrate that mRNA-derived VRC01 protects from HIV-1 challenge [267]. mRNA encoding a reporter protein was utilized as control. mRNA-LNPs were administered 24 h prior to challenge with one of two HIV-1 isolates. In the authors’ first model, a VRC01 mRNA dose of 0.35 mg/kg was ineffective, but 0.7 mg/kg already reduced viral RNA copies in the plasma to undetectable levels as assessed by quantitative real-time polymerase chain reaction (qRT-PCR). The latter dose is well below the 10–20 mg/kg doses that are typically used for prophylactic immunization with recombinant antibody in humanized mice to reach therapeutic concentrations [272, 273]. However, the authors did not titrate the dose of recombinant VRC01 but used a 28 mg/kg dose as control which was sufficient to completely eradicate viral RNA copies in the plasma. mRNA efficacy could be also demonstrated in the second mouse challenge model.

To show in vivo efficacy of mRNA-mediated BiTE expression, Stadler et al. implanted tumor cells subcutaneously in immunodeficient NSG mice [264]. About 1 week before mRNA treatment, human PBMCs were engrafted into these animals. 3 µg of BiTE mRNA (approx. 0.15 mg/kg) given three times with an interval of 1 week could eliminate tumors entirely. In contrast, tumors progressed in control animals that received mRNA encoding a reporter protein. The recombinant BiTE required three injections per week and a total of ten injections of 4–7 µg each to obtain a comparable antitumor effect as with BiTE mRNA. The need for a more frequent administration corroborated the previous finding that mRNA substantially improved antibody plasma half-life.

Due to the diversity of antibodies included in their study, Thran et al. utilized various disease models for demonstrating therapeutic efficacy [268]. In contrast to all other studies, the authors applied mRNA encoding irrelevant antibodies instead of a reporter protein as control. A 40 µg dose (approx. 2 mg/kg) of antibody mRNA could protect mice from challenges with either rabies virus or botulinum toxin. In the intoxication model, mRNA was proven to be equally protective as recombinant antibody. However, mice received approximately 0.1 mg/kg of recombinant VNA compared to approximately 2 mg/kg of mRNA. Based on protein expression levels from mRNA dose titrations, lower doses than 2 mg/kg may still confer full protection but this remains hypothetical, since the authors did not conduct an mRNA dose titration in their challenge model. Notably, mRNA was effective in pre- as well as in post-exposure settings. The latter is important for some indications of passive immunization and confirms the aforementioned rapid onset of antibody expression. The post-exposure scenario for botulinum toxin requires very rapid availability of neutralizing antibodies. Whereas recombinant protein can act immediately after administration, mRNA needs more time to provide the antibody. Hence, it may well be that in such instances higher doses of mRNA than of protein are required, not for obtaining the same peak level but for reaching meaningful titers in a timely manner. In a further model, Thran et al. evaluated their mRNA approach with respect to anti-tumor efficacy. Using a disseminated tumor model for Rituximab, they showed efficient tumor growth control with injections of 50 µg (approx. 2.5 mg/kg) of Rituximab mRNA twice a week. Higher doses (200 µg, approx. 10 mg/kg) of recombinant Rituximab were less potent. This finding is reminiscent of results of Drew Weissman’s group and contrasts those of Ugur Sahin and colleagues who required similar doses of mRNA and recombinant protein (but less frequent dosing with mRNA) to obtain equivalent therapeutic effects [264, 267]. Notably, the difference among studies may be related to the use of IgG antibodies on the one hand and a scFv_2_ protein on the other hand. Moreover, the irrelevant antibody control used by Thran et al. appeared to have a slight unspecific anti-tumor effect. It may have contributed to the superiority of mRNA compared to recombinant protein regarding dosing. Amongst other explanations, the potential unspecific effect may be due to an mRNA-LNP-independent response to repeated treatment or may be the consequence of a weak and transient cytokine response observed after mRNA-LNP administration. However, the phenomenon was not investigated further.

#### Tolerability of mRNA-based passive immunization

In line with previous reports, Pardi et al. confirmed the importance of highly purified IVT mRNA. In combination with LNPs, only modification with *N*1-methyl-pseudouridine plus chromatographic purification was sufficient to avoid cytokine release by innate immune activation [267]. For this analysis, however, the authors deployed an mRNA encoding a different protein than the VRC01 antibody which had been used for in vivo expression and efficacy experiments. Tolerability of mRNA-LNPs was also addressed by repeated mRNA treatments. Translation of VRC01 mRNA was not compromised over time, but the analysis was conducted in immunodeficient NSG mice. To overcome this caveat, the authors complemented their study by repetitive treatment of immune competent BALB/c mice. To this end, they switched to an endogenous protein, since human VRC01 may be recognized as foreign and thus elicit an immune response. Again, mRNA injections did not lose efficacy over time. However, the authors also changed the formulation (TransIT instead of LNP) as well as the route of administration (intraperitoneal instead of IV) compared to the use of VRC01 mRNA. Hence, evidence for immune silence and overall tolerability of VRC01 mRNA in LNPs is just circumstantial yet.

Stadler et al. did not observe any liver toxicity upon treatment with mRNA in TransIT according to liver enzyme analyses [264]. Moreover, BiTE mRNA administration did not elevate murine cytokines such as IFNα and TNFα above background in plasma. Likewise, analysis of systemic human cytokine release from engrafted PBMCs did not show any unspecific T cell activation. As opposed to modified and HPLC-purified mRNA, preparations without nucleotide modification and chromatographic purification induced detectable levels of murine cytokines. Similar to the Weissman group, the authors also assessed the tolerability of formulated mRNA by repeated injections. Administrations did not lose efficacy over time, but as in the corresponding Weissman experiment immunodeficient NSG mice were used.

Using chemically unmodified mRNA formulated in LNPs, Thran et al. did not observe any liver toxicity in histopathological analyses [268]. Only a few animals developed an ADA response which was dependent on encoded antibody and was, thus, no intrinsic consequence of treatment with mRNA-LNP. In addition, treatment appeared to elicit a transient weak cytokine release which, however, neither suppressed antibody expression nor induced adverse effects. Since there are ample differences among studies on mRNA-mediated antibody expression and no detailed analyses of the issue, the role of mRNA, LNP, and/or encoded antibody/protein in cytokine induction remains elusive. An earlier study on erythropoietin showing the absence of any appreciable immunostimulation suggests that the use of chemically unmodified instead of modified mRNA is not the decisive parameter [218].

## Conclusions

Quite a few in vivo studies provided compelling evidence for the principle feasibility of mRNA-based immunotherapies. As discussed above, challenges and open questions regarding adoptive T cell transfer are less related to the mRNA and its formulation or transfection but more of fundamental character. In contrast to ex vivo loading of cells, mRNA-mediated antibody expression is strongly affected by body size. Thus, while there are now convincing efficacy data in diverse small rodent models, the translation to larger animals and finally humans has still to be demonstrated. First data suggest that substantial expression can be obtained in small NHPs. However, the utilized LNPs appeared to lose some efficacy when switching from mouse to NHP. Hence, the development of human therapies may perhaps require further advancements of the mRNA technology as well as primate-specific formulations with improved efficacy. In addition, tolerability of formulations has to be analyzed further and in more depth in the future. For instance, repeated dosing of nanoparticles can induce complement activation-related pseudoallergy (CARPA) [274]. However, this can in principle be counteracted by optimization towards better biocompatibility. In case of LNPs, fast degradation was shown to be particularly important [231, 232, 269].

While antibodies for cancer treatment were initially developed for IV administration, there is a trend towards subcutaneous injections today. For instance, Rituximab was initially formulated for IV infusion which is typically administered over a period of 1.5–6 h [275]. This treatment schedule poses a substantial burden to patients as well as the healthcare system. Thus, a formulation which reduces the time and required resources would be advantageous. To meet these goals by subcutaneous administration, the antibody solution was concentrated 12-fold [276, 277]. Since this volume was still too large for subcutaneous injection, Rituximab was co-formulated with human hyaluronidase which limits swelling and associated pain by increasing the dispersion and absorption of co-administered substances [276, 278]. Now, median administration time for Rituximab using the subcutaneous route is 6 min. As a consequence, antibody immunotherapy with mRNA does not only require competitive efficacy and costs but also routes of administration to become a viable alternative to recombinant proteins. Although other routes than IV have been shown to be possible for mRNA, there are still a few open questions to be addressed by future studies.

Where are the advantages of using mRNA for antibody immunotherapies? Compared to DNA it may be primarily the safety aspect. Concerning recombinant proteins various points matter. As reviewed above, mRNA provides benefits as to the pharmacokinetics when short-lived antibodies such as scFv, (bi)-scFv_2_ or VNA are used. Moreover, solving the challenges of antibody cocktails may be easier using mRNA. Different mRNA sequences are much more similar with respect to their physicochemical characteristics than different proteins are. Hence, producing a cocktail may be less demanding for mRNA compared to protein. However, co-delivery and thus co-expression implicates the risk of antibody chimerism and thus requires specific solutions such as knob-into-hole concepts [68]. Last but not least, while proteins are difficult to deliver directly through the cell membrane [279], mRNA-mediated protein expression makes a large number of potential intracellular targets accessible to antibody immunotherapy. Particularly single-chain and single-domain formats are amenable to functional expression in the cytosol and thus suited as intrabodies, since they are less dependent on disulfide bond formation [280, 281]. The value of targeting intracellular proteins has already been demonstrated by various studies. A bispecific scFv could restore p53 function in mutant p53 colon cancer cells and trapping CCR5 in the ER via an intrabody reduced HIV cell entry [282, 283]. Support for the potential of intrabodies as therapeutics also comes from further work in the field of oncology or neurodegenerative diseases [284–286]. Although it has been recently demonstrated that even a full antibody can be delivered into cells in vivo [287], it has been recognized that fusions of cell-penetrating peptides (CPPs) and macromolecules are often trapped in endosomes instead of being released to the cytoplasm [288]. In contrast, nucleic acids including mRNA can be very efficiently transfected into cells, making them ideal for the delivery of intrabodies. However, while LNPs provide very efficient solutions for systemic delivery to the liver, formulations for routing mRNA to other tissues are scarce today. With the recent burst of publications on successful applications of mRNA-based antibody immunotherapy it is likely that there will be much to follow in the near future.

## Abbreviations

AAV: Adeno-associated virus
ADA: Anti-drug antibodies
ARCA: Anti-reverse cap analog
bi-(scFv)_2_: Bivalent single chain variable fragment
BiTE: Bi-specific T cell engager
CAR: Chimeric antigen receptor
CD: Cluster of differentiation
CTL: Cytotoxic T lymphocyte
DC: Dendritic cell
DNA: Deoxyribonucleic acid
*E. coli*: *Escherichia coli*
Fab: Fragment antigen binding
Fc: Fragment crystallizable
FcRn: Neonatal Fc receptor
FPLC: Fast protein liquid chromatography
GMP: Good manufacturing practice
HIV: Human immunodeficiency virus
HPLC: High performance liquid chromatography
IFIT: Interferon induced protein with tetratricopeptide repeats
IgG: Immunoglobulin G
IRES: Internal ribosome entry site
IVT: In vitro transcription
LNP: Lipid nanoparticle
mAb: Monoclonal antibody
MHC: Major histocompatibility complex
mRNA: Messenger ribonucleic acid
NSG: NOD (non-obese diabetic) scid gamma
ORF: Open reading frame
Poly(A): Polyadenylic acid
PKR: Protein kinase R
RIG-I: Retinoic acid inducible gene I
scFv: Single chain variable fragment
TAA: Tumor-associated antigen
TCR: T-cell receptor
TLR: Toll-like receptor
UTR: Untranslated region
VEGF: Vascular endothelial growth factor
V_H_: Heavy-chain variable region
V_H_H: Heavy-chain-only V_H_ domain
VNA: V_H_H-based neutralizing agent
